# A multi-target therapeutic potential of *Prunus domestica* gum stabilized nanoparticles exhibited prospective anticancer, antibacterial, urease-inhibition, anti-inflammatory and analgesic properties.

**DOI:** 10.1186/s12906-017-1791-3

**Published:** 2017-05-23

**Authors:** Nazar Ul Islam, Raza Amin, Muhammad Shahid, Muhammad Amin, Sumera Zaib, Jamshed Iqbal

**Affiliations:** 1 Department of Pharmacy, Sarhad University of Science and Information Technology, Peshawar, 25000 Pakistan; 2 Institute of Chemical Sciences, University of Peshawar, Peshawar, Pakistan; 3 Department of Chemistry, Islamia College University, Peshawar, Pakistan; 4 Centre for Advanced Drug Research, COMSATS Institute of Information Technology, Abbottabad, Pakistan

**Keywords:** Pharmaceutical gums, Gum-loaded nanoparticles, Multi-targeted therapeutics, Nano-drug delivery, Nano-stability, Cancer, Antimicrobial, Enzyme inhibition, Inflammation, Pain

## Abstract

**Background:**

Phytotherapeutics exhibit diverse pharmacological effects that are based on the combined action of a mixture of phytoconstituents. In this study, *Prunus domestica* gum-loaded, stabilized gold and silver nanoparticles (Au/Ag-NPs) were evaluated for their prospective anticancer, antibacterial, urease-inhibition, anti-inflammatory, and analgesic properties.

**Methods:**

Au/Ag-NPs were biosynthesized and characterized with UV-Vis, FTIR, SEM, EDX, and XRD techniques. The effect of gum and metal ion concentration, reaction temperature, and time on the synthetic stability of nanoparticles was studied along with their post-synthetic stability against varying pH and salt concentrations, long-term storage and extremes of temperature. Nanoparticles were tested for anticancer (HeLa cervical cancer cells), antibacterial (*Staphylococcus aureus*, *Escherichia coli,* and *Pseudomonas aeruginosa*), urease inhibition (jack-bean urease), anti-inflammatory (carrageenan-induced paw edema), and antinociceptive (abdominal constriction response) activities.

**Results:**

The nanoparticles were mostly spherical with an average particle size between 7 and 30 nm (Au-NPs) and 5–30 nm (Ag-NPs). Au/Ag-NPs maintained their colloidal stability and nanoscale characteristics against variations in physicochemical factors. Au/Ag-NPs have potent anticancer potential (IC_50_ = 2.14 ± 0.15 μg/mL and 3.45 ± 0.23 μg/mL). Au/Ag-NPs selectively suppressed the growth of *S. aureus* (10.5 ± 0.6 mm, 19.7 ± 0.4 mm), *E. coli* (10 ± 0.4 mm, 14.4 ± 0.7 mm), and *P. aeruginosa* (8.2 ± 0.3 mm, 13.1 ± 0.2 mm), as well as showed preferential inhibition against jack-bean urease (19.2 ± 0.86%, 21.5 ± 1.17%). At doses of 40 and 80 mg/kg, Au-NPs significantly ameliorated the increase in paw edema during the 1st h (*P* < 0.05, *P* < 0.01) and 2–5 h (*P* < 0.001) of carrageenan-induced inflammation compared to the 200 and 400 mg/kg doses of *P. domestica* gum (*P* < 0.05, *P* < 0.001). At similar doses, Au-NPs also significantly abolished (*P* < 0.01) the tonic visceral, chemically-induced nociception, which was comparable to that of *P. domestica* gum (200 mg/kg; *P* < 0.05, 400 mg/kg; *P* < 0.01).

## Conclusions

The *Prunus domestica* gum-integrated nanoparticles have multi-target therapeutic capabilities and thus possess an advantage in combating multigenic diseases that affect multiple tissues or cell types.

## Background

Drugs designed to act against individual molecular targets cannot usually combat multigenic diseases or diseases that affect multiple tissues or cell types. Combination drugs that impact multiple targets simultaneously are better at controlling complex disease systems, are less prone to drug resistance, and are the standard of care in many important therapeutic areas [1]. The multiple target therapeutic approach increasingly is used to treat many types of diseases, including AIDS, atherosclerosis, cancer, and depression [2]. The low affinity of multi-target drugs is more likely to induce synergistic therapeutic effects by the combination of various mechanistic actions. The therapeutic efficacy of phytotherapy is based on the combined action of a mixture of constituents and offers new treatment opportunities [3].

Compared to conventional systems of traditional medicine, the incorporation of the nano-traditional concept has several advantages, including (1) improvement of the biological availability and therefore saves the limited resources of the Materia Medica; (2) strengthening of the target-oriented therapeutic effects; (3) provide pharmaceutical preparation choices; and (4) promote the standardization and internationalization of the drug preparation. This concept has been successfully implemented in the Chinese Materia Medica and has shown many advantages [4]. The combination of nanotechnology with traditional herbal medicine therefore provides a very useful tool in designing future herbal medicine with an improved bioavailability profile and less toxicity. This new approach is increasing the interest of a number of scientists to improve and to accelerate the joint drug discovery and development of novel nano-delivery systems for herbal extracts [5].

*Prunus domestica* L. (family *Rosaceae*) is a shrubby, deciduous, small tree cultivated at high altitude. The fruit of *P. domestica* is used medicinally for the treatment of leukorrhea, irregular menstruation, and debility following miscarriage. The fruit has been shown to lower low-density lipoprotein (LDL) cholesterol in human plasma [6] as well as plasma and liver lipids in rats [7], prevent and improve ovariectomy-induced hypercholesterolemia in rats [8] and bone mineral density loss in postmenopausal women [9], possesses antiemetic action against apomorphine-induced emesis in dogs [10], and has antinociceptive efficacy in rats [11] along with potent antibacterial activity [12]. *P. domestica* dried fruit contains large amounts of antioxidant constituents, such as neochlorogenic acid (3-O-caffeoylquinic acid), chlorogenic acid (5-O-caffeoylquinic acid), cryptochlorogenic acid (4-O-caffeoylquinic acid), (+)-abscisic acid (5), (+)-β-D-glucopyranosyl abscisate (6), (6S,9R)-roseoside (7), and two lignan glucosides [(+)-pinoresinol mono-β-D-glucopyranoside (8) and 3-(β-D-glucopyranosyloxymethyl)-2 -(4-hydroxy-3-methoxyphenyl)-5 -(3-hydroxypropyl)-7 -methoxy-(2R,3S) -dihydrobenzofuran (9)] [13, 14]. In addition, the fruit contains flavonols (myricetin, quercetin, and kaempferol), carbohydrates (fructose, sucrose, glucose, sorbitol), organic acids (citric acid, malic acid), vitamins (α-tocopherol, γ-tocopherol, β-carotene), and minerals (sodium, potassium, magnesium, calcium, iron, zinc) [15]. *P. domestica* fruit-extract has been used as a reducing agent for the efficient synthesis of gold nanoparticles and showed a dose-dependent catalytic activity [16].

Gums are water-soluble polysaccharides (including modified polysaccharides), which produce viscous aqueous systems, generally at low concentrations. The gums are apparently not normal products of plant metabolism, but probably are more or less pathological products formed by plants when injured or diseased or under adverse climatic conditions [17]. Generally, plant gum exudates contain galactose, arabinose, rhamnose, uronic acids, galacturonic acid, protein, Ca and Mg as major structure constituents as well as, glucose, xylose, mannose, protein, and fat as minor constituents [18]. Natural gums along with mucilages constitute a structurally diverse class of biological macromolecules with a broad range of physicochemical properties, which are widely used for various applications in pharmacy and medicine [19]. There is a huge scope of natural gums as a novel natural polymer for the development of different drug delivery systems. In this study we evaluated the *P. domestica* gum-loaded, stabilized gold and silver nanoparticles for their prospective in vitro anticancer, antibacterial, and urease inhibition activities. Moreover, the *P. domestica* gum-loaded gold nanoparticles were assessed for in vivo anti-inflammatory and analgesic properties. Gold nanoparticles show several features that make them well suited for biomedical applications including their ease of synthesis, high surface area, stability and low inherent toxicity [20, 21], compared to silver nanoparticles, which are toxic to mammalian cells and produce adverse-effects in different organs [22].

## Methods

### Materials

Tetrachloroauric acid trihydrate (HAuCl_4_.3H_2_O, 99.5%) and silver nitrate (AgNO_3_, 99.9%) were purchased from Merck, Germany. *Prunus domestica* fresh gum was purchased from the local market in April 2013 and was formally identified (RA-85) prior to its use by Prof. Dr. Samen Jan of Department of Botany, Islamia College University, Peshawar, Pakistan. Water was purified through a Milli-Q-SP ultra pure water purification system.

### Synthesis of gold and silver nanoparticles

The *P. domestica* gum-mediated biosynthesis of gold and silver nanoparticles was carried out by utilizing the stock solutions of tetrachloroauric acid trihydrate/silver nitrate and *P. domestica* gum at concentrations of 1 mM and 0.5% *w*/*v*, respectively. These solutions were centrifuged at 5000×g for 10 min to remove bulk impurities. The aqueous solutions of tetrachloroauric acid and silver nitrate were reduced by mixing with 0.5% *P. domestica* gum solution in differing ratios and stirred gently at temperatures of 20, 40, 60 and 80 °C. The optimized product having surface plasmon resonance (SPR) at 555 nm for the gold nanoparticles was obtained by mixing 8 mL of tetrachloroauric acid solution (1 mM) and 5 mL of 0.5% *w*/*v P. domestica* gum solution at a temperature of 80 °C and a reaction time of 5 h. Similarly, the optimized silver nanoparticles having SPR at 450 nm were obtained by use of 20 mL of silver nitrate solution (1 mM) and 8 mL of 0.5% *w*/*v P. domestica* gum solution at a temperature of 80 °C.

### Characterization of gold and silver nanoparticles

*P. domestica* gum-loaded gold and silver nanoparticles were characterized on a double beam UV-Vis spectrophotometer (Lambda 25, Perkin Elmer) in the spectral range of 250–800 nm, FTIR spectrophotometer (Prestege-21 Shimadzu, Japan), scanning electron microscope (SEM, JSM-5910, England), energy dispersive X-ray spectrometer (EDX, INCA-200, England), X-ray diffractometer (XRD, RX-III, Shimadzu, Japan) at 40 kV and 30 mA with CuKα radiation (λ = 0.1542 nm), and atomic absorption spectrophotometer (AAS-700 Perkin Elmer, USA). Thermo gravimetric analysis (TGA) was performed on a Diamond TG/DTA Perkin Elmer, USA thermogravimetric analyzer.

### Assessment of stability

The effect of gum concentration on the synthesis of gold and silver nanoparticles was studied by heating different concentrations (0.1–0.5%) of gum solutions containing 1 mM of tetrachloroauric acid and silver nitrate solutions, respectively, for 1 h. The effects of gold or silver ions were evaluated by changing their concentration from 1 to 5 mM and then heating at 80 °C for 3 h. The thermal stability was studied by keeping the nanoparticles solution at 20, 40, 60, 80 °C, each for 3 h. The effect of varying reaction time (1–5 h) was assessed with 0.5% gum at 1 mM gold or silver salt solution. The salt stability was checked by adding 20, 40 and 60 μL of sodium chloride solution (1 M) to 3 mL colloidal solution of gold and silver nanoparticles under continuous mixing for 3 h. The resistance to varying pH conditions was measured at different pH values (2–3, 4–5, 6–7, 8–9, 10–11, 12–13) by drop-wise addition of 1 M HCl or NaOH solution. The long term stability was estimated by keeping the nanoparticles at room temperature for eight months. The extreme thermal stability was evaluated by heating the nanoparticles at 100 °C for 30 min.

### In vitro biological assays

#### Cytotoxicity assay

The HeLa cervical cancer cell line (HeLa cells) was cultured in RPMI-1640, having heat-inactivated fetal bovine serum (10%), glutamine (2 mM), pyruvate (1 mM), 100 U/mL penicillin, and 100 μg/mL streptomycin, in T-75 cm^2^ sterile tissue culture flasks in a 5% CO_2_ incubator at 37 °C. For experiments, 96-well plates were used for growing HeLa cells by inoculating 5 × 10^4^ cells per 100 μL per well, and plates were incubated at 37 °C for 24 h in a humidified atmosphere containing 5% CO_2_. Within 24 h, a uniform monolayer was formed, which was used for experiments. To perform the cytotoxicity assay, a previously described method [23] was adapted with small modifications [24]. Briefly, cells were cultured in different 96-well plates for 24 h. Initially, 1 mg/mL of *P. domestica* gum solution and the nanoparticles were inoculated in test wells. Further these solutions screened for different concentrations (1 mg/mL – 1 ng/mL) were inoculated in test wells. Cisplatin was used as a positive control. The well containing culture media with cells having no compound or drug was taken as blank. All the plates were then incubated for 48 h. After that, cells were fixed with 50 μL of 50% ice cold trichloroacetic acid solution (TCA), and plates were incubated at 4 °C for 1 h. Subsequently, plates were washed five times with phosphate-buffered saline (PBS) and air dried. Fixed cells were further treated with 0.4% *w*/*v* sulforhodamine B dye (prepared in 1% acetic acid solution) and left at room temperature for 30 min. The plates were rinsed with 1% acetic acid solution and allowed to dry. In order to solubilize the dye, the dried plates were treated with 10 mM Tris base solution for 10 min at room temperature. The absorbance was measured at 490 nm subtracting the background (blank) measurement at 630 nm [25]. All experiments were performed in triplicate. The IC_50_ values of potential inhibitors (≥50%) were determined with the help of the non-linear regression analysis program of GraphPad Prism 5.0 Software Inc., San Diego, CA, USA [24].

#### Urease inhibition assay

Urease inhibition activity of the synthesized compounds was determined by the indophenol method [26] with small modifications [27]. Reaction mixtures comprised of 40 μL of buffer (100 mM urea, 0.01 M K_2_HPO_4_, 1 mM EDTA and 0.01 M LiCl_2_, pH 8.2) and 10 μL of jack-bean urease enzyme (5 U/mL) were incubated with 10 μL of *P. domestica* gum solution and the nanoparticles (1 mg/mL) at 37 °C for 30 min in 96-well plates. Urease inhibitory activity was calculated by the indophenol method based on the production of ammonia. The phenol reagent (40 μL, 1% *w*/*v* phenol, 0.005%, *w*/*v* sodium nitroprusside) and alkali reagent (40 μL 0.5% *w*/*v* NaOH, 0.1% active chloride NaOCl) were added to each well, and, after 10 min of incubation at 37 °C, the absorbance was measured at 630 nm with a microplate reader (Bio-TekELx 800™, Instruments, Inc. Winooski, VT, USA). Thiourea was used as the standard inhibitor. All experiments were performed in triplicate. The percent inhibition was calculated according to the equation,

Percent inhibition = 100 – [absorbance of nanoparticles/ absorbance of control] × 100.

#### Antibacterial assay

The antibacterial activity of *P. domestica*-loaded gold and silver nanoparticles was evaluated against Gram-positive [*Staphylococcus aureus* (ATCC 25923)] and Gram-negative [*Escherichia coli* (ATCC 25922), and *Pseudomonas aeruginosa* (ATCC 27853)] strains of bacteria, by the disc diffusion method. Three independent experiments were carried out for each bacterial strain with streptomycin as the positive control. Au/Ag-NPs (5 μg) were dissolved in DMSO and incubated at 30 °C for 24 h. The final DMSO concentration was kept below 1%.

### In vivo biological assays

#### Animals

BALB/c mice of either sex weighing 25–30 g were purchased from the National Institute of Health (NIH), Islamabad, for the in vivo experiments. The animals were acclimatized at 22 ± 2 °C for one week prior to experiments. The animals have free access to food and water during this period. Prior to experiments, the animals were fasted for 2 and 4 h, respectively, for antinociceptive and anti-inflammatory assay. All experimental procedures on animals were performed in accordance with the NIH guidelines for the care and use of laboratory animals. The experimental protocols conformed to the Animal Research: Reporting In Vivo experiments (ARRIVE) guidelines. The study was approved by the Graduate Studies Committee (GSC) of the Institute of Chemical Sciences (ICS), University of Peshawar (reference letter number: 942–51/ICS). Animals were randomly assigned to different treatment groups, with each group consisting of six animals.

#### Antinociceptive assay

The antinociceptive efficacy afforded by *P. domestica* gum-loaded gold nanoparticles against tonic visceral chemically-induced nociception was determined by the acetic acid-induced abdominal constriction assay [28]. All drugs were dissolved in normal saline and were administered through an oral gavage tube, except diclofenac sodium, which was given as an intraperitoneal injection. Animals were randomly divided into six groups, with each group containing 6 mice. Group I received normal saline and served as control. Group II was administered with the positive control, diclofenac sodium (50 mg/kg, i.p). Group III and IV received *P. domestica* gum at 200 and 400 mg/kg, while group V and VI were treated with *P. domestica* gum-loaded gold nanoparticles at doses of 40 and 80 mg/kg, respectively. After 30 min of drugs treatment, the animals were injected with 1% acetic acid (10 mL/kg, i.p) and the abdominal writhes were counted for 20 min.

#### Anti-inflammatory assay

The inhibitory effect produced by *P. domestica* gum-loaded gold nanoparticles against a phlogistic agent-mediated paw swelling was evaluated by the carrageenan induced paw edema assay [29]. Animals were divided into seven groups, with each group containing 6 mice. *P. domestica* gum and its gold nanoparticles were administered by an oral gavage tube. Diclofenac sodium was used as positive control and was injected i.p at a dose of 50 mg/kg. After 30 min of treatment, all animals were challenged with 50 μL of 1% solution of carrageenan, injected into the plantar surface of the left hind paw. The anti-inflammatory effect was evaluated by measuring the paw volume of each animal with a digital plethysmometer after each hour of the 5 h study duration.

### Statistical analysis

Data were expressed as mean ± SD or SEM. Statistical analysis was done by one-way ANOVA followed by Dunnett’s or Tukey’s post hoc test where appropriate with GraphPad Prism 5.0 (GraphPad Software Inc., San Diego, CA, USA).

## Results

### Characterization of synthesized gold and silver nanoparticles

#### UV-Vis spectroscopy

**Fig. 1:**
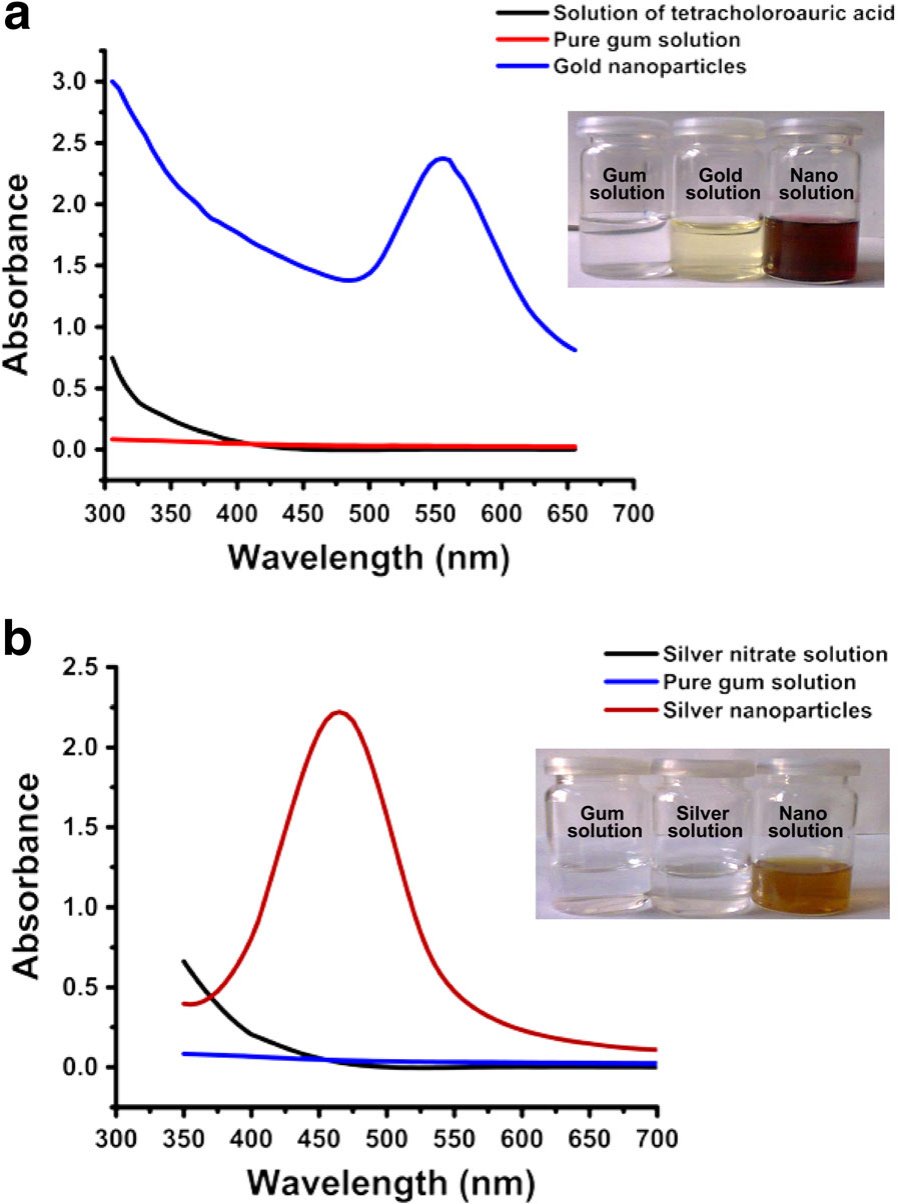
UV-Vis spectra of *P. domestica* gum-loaded gold (**a**) and silver (**b**) nanoparticles. The inset photos show the corresponding color of the biosynthesized nanoparticles solutions

UV-Vis absorption spectra of *P. domestica* gum-loaded gold and silver nanoparticles solutions were recorded against *P. domestica* gum and pure metal salt solutions. The typical SPR peaks for gold and silver nanoparticles were obtained at 555 and 450 nm, respectively, however, no SPR peaks were observed for the pure chlorauric acid, silver nitrate, and gum solutions. The biosynthesis of gold and silver nanoparticles were further confirmed from the appearance of wine red and light yellow color respectively, while the pure gum, chloroauric acid, and silver nitrate solutions appeared colorless (Fig. 1).

#### FTIR

**Fig. 2:**
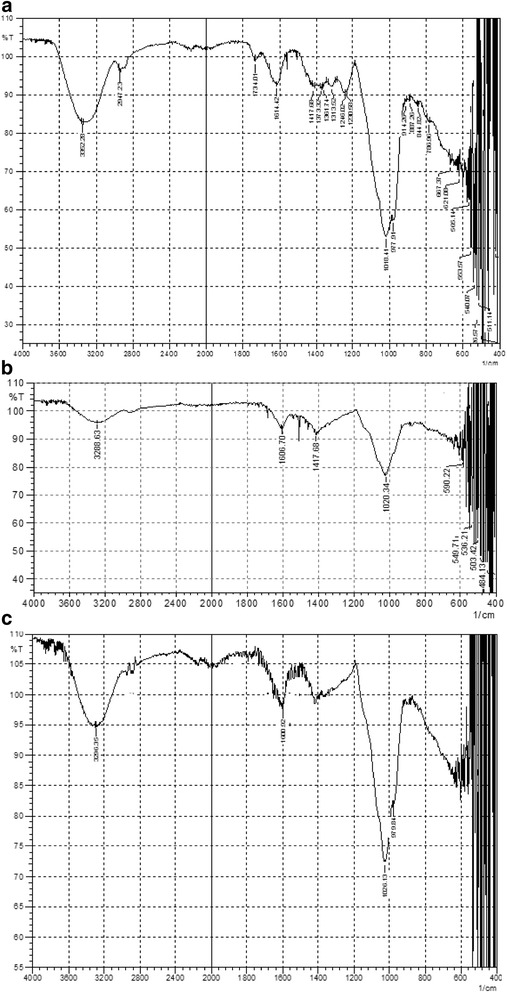
FTIR spectra of *P. domestica* gum (**a**) and *P. domestica* gum-loaded gold (**b**) and silver (**c**) nanoparticles

*P. domestica* gum has mainly polysaccharide compounds, which, along with glucuronic acid and its 4-O-methyl ether having O-H, −COOH, hemiacetal and ether groups, showed a broad O-H stretching band at 3352 cm^−1^ (Fig. 2a). A weak peak at 2947 cm^−1^ was due to C-H stretching. The peak observed at 1734 cm^−1^ corresponds to the C = O stretching of -COOH. Similarly, a strong peak at 1614 cm^−1^ can be attributed to the conjugated C = C bond stretching, which might be due to the presence of flavonoids and carotenoids. Coupled vibrations due to C-O stretching and O-H deformations were observed at 1417 and 1373 cm^−1^. The strong peak at 1018 cm^−1^ corresponds to C-O stretching vibrations. In case of the gold nanoparticles (Fig. 2b), the O-H bond stretching shifted from 3352 cm^−1^ to the lower frequency of 3288 cm^−1^, and this shows the involvement of the OH groups during the process of reduction and capping. A sharp carbonyl carbon peak at 1734 cm^−1^ disappeared completely, thus demonstrating the participation of carboxylic acid groups in nanoparticle formation as a reducing and capping agent. The bending vibrations at 1373, 1361, 1313, 1246, and 1220 cm^−1^ also disappeared due to possible attraction by the gold nanometal. A shift in the C-O acyclic bond stretching from 1018 to 1020 cm^−1^ can be attributed to attraction by gold metal during the capping process. Almost similar FTIR spectrum was observed for the *P. domestica* gum-loaded silver nanoparticles (Fig. 2c).

#### SEM

**Fig. 3:**
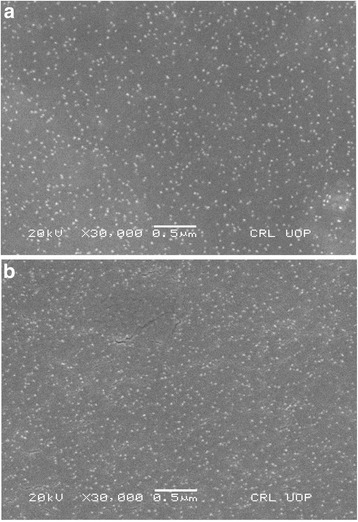
Scanning electron microscopic images of *P. domestica* gum-loaded gold (**a**) and silver (**b**) nanoparticles

*P. domestica* gum-loaded gold and silver nanoparticles were in the size range of 7–30 and 5–30 nm, respectively, with most particles were spherical in shape, but a small number of anisotropic nanostructures, such as nanotriangles, a few nanorods, and hexagonal and polygonal nanoprisms were also present (Fig. 3). The uniform distribution of gold and silver nanoparticles indicates the complete stabilization of nanoparticles by capping agents, while the large sizes and anisotropic shapes of some particles might be due to the aggregation of the smaller nanoparticles.

#### EDX and XRD

**Fig. 4:**
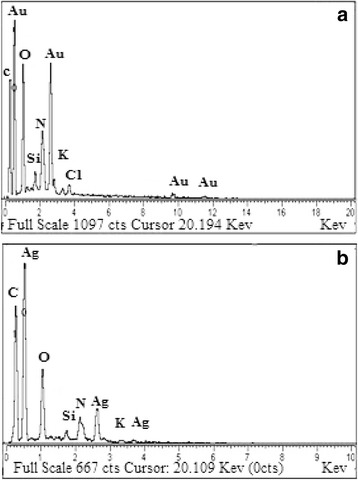
EDX spectra of *P. domestica* gum-loaded gold (**a**) and silver (**b**) nanoparticles

The EDX analysis (Fig. 4) indicated the presence of gold and silver in the *P. domestica* gum-loaded gold and silver nanoparticles. Strong signals were observed from Au atoms in the gold nanoparticles at approximately 0.6 and 2.6 keV, while a weak signal was observed at 9.7 keV. In the case of silver nanoparticles, strong signals were observed at 0.6 and 2.6 keV, while a weak signal was observed at 3.7 keV. The appearance of the Si signal corresponds to the use of a silicon grid in the EDX analysis. The signal for N indicates the presence of nitrogen-containing organic compounds, while the signals for K and Mg correspond to the X-ray emission from different bio-molecules of the gum. Similarly, the other strong signals for C and O also were due to the presence of bio-organic molecules capping the gold and silver nanoparticles. The presence of the Cl atom in the EDX spectra of gold nanoparticles was due to its presence in the tetrachloroauric acid trihydrate molecule. The appearance of elemental Au and Ag in the EDX analysis supports the XRD results, which provides confirmation of the reduction of metal cations to elemental form.

**Fig. 5:**
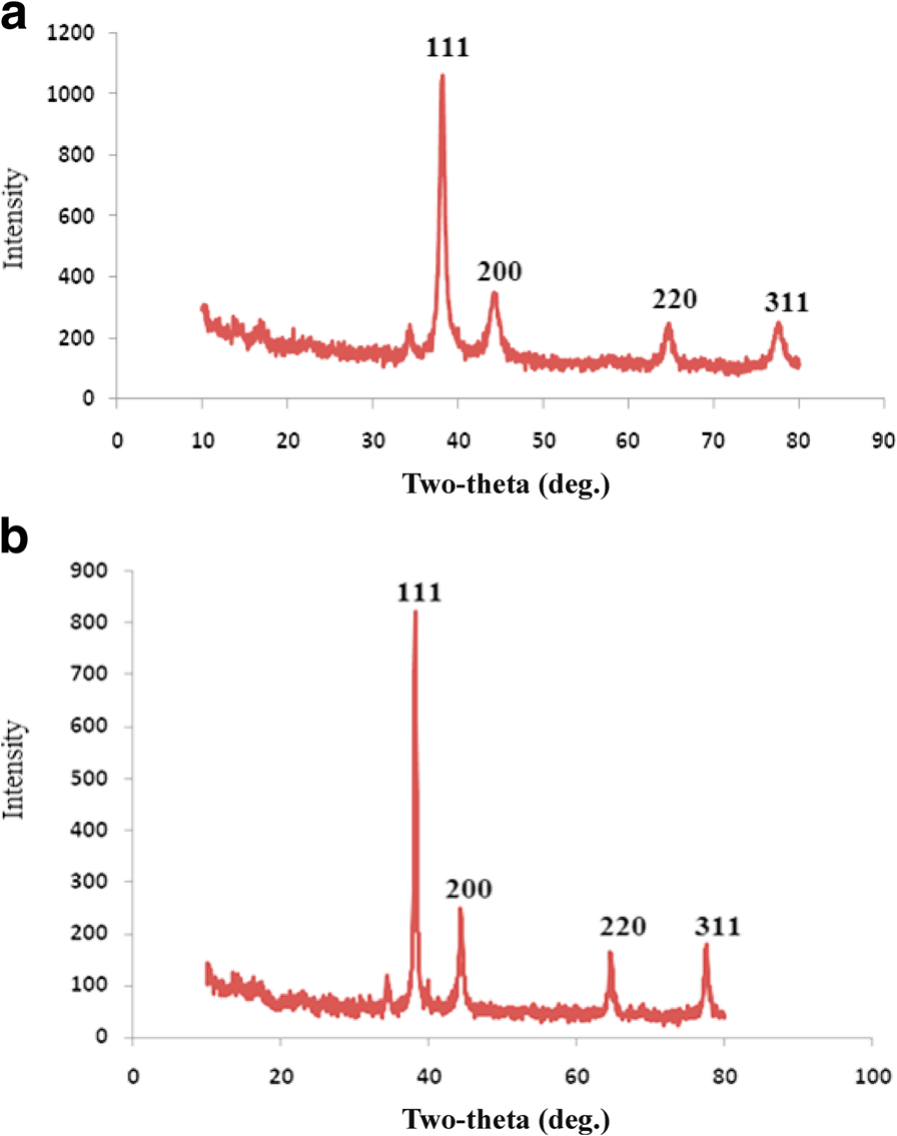
XRD patterns of *P. domestica* gum-loaded gold (**a**) and silver (**b**) nanoparticles

In the XRD spectrum, no peaks were observed for the *P. domestica* gum, thus indicating their non-crystalline (amorphous) nature. However, the XRD patterns of gum-loaded gold and silver nanoparticles have characteristic peaks, indicating their crystalline nature. The gold nanoparticles (Fig. 5) exhibited characteristic peaks at scattering angles (2 Ø) of 38.431, 44.623, 64.531, 77.781 that can be indexed respectively to the (111), (200), (220), (311) Bragg’s reflections of the face-centered cubic (FCC) structure of metallic gold similar to the Joint Committee on Powder Diffraction Standards (JCPDS) file no: ICDD-PDF2, revealing that the synthesized Au-NPs are of pure crystalline gold. In the case of silver nanoparticles, the characteristic diffraction peaks of FCC metallic silver phase (4–0784) were observed at 38.21, 44.39, 64.62 and 77.59. Furthermore, no peaks similar to other crystalline phases were observed, indicating the high purity of the resultant nanoparticles. The diffraction peak at 38^o^ was the only intense peak among the observed peaks for both the gold and silver nanoparticles. In addition, the ratio between the (2 2 0) and (1 1 1) peaks was lower than the standard value (0.1 vs. 0.4). The mean particle diameters of the gold and silver nanoparticles were calculated from the XRD data. Diameters were derived from the Debye Scherrer eq. (D = *k* λ/ β½ cosθ). This equation exploits the reference peak width at an angle θ, where λ is the X-ray wavelength (1.5418), β½ is the width of the XRD peak at half height and *k* is the shape factor. Average particle size was around 20 and 17 nm for gold and silver nanoparticles, respectively.

#### TGA

**Fig. 6:**
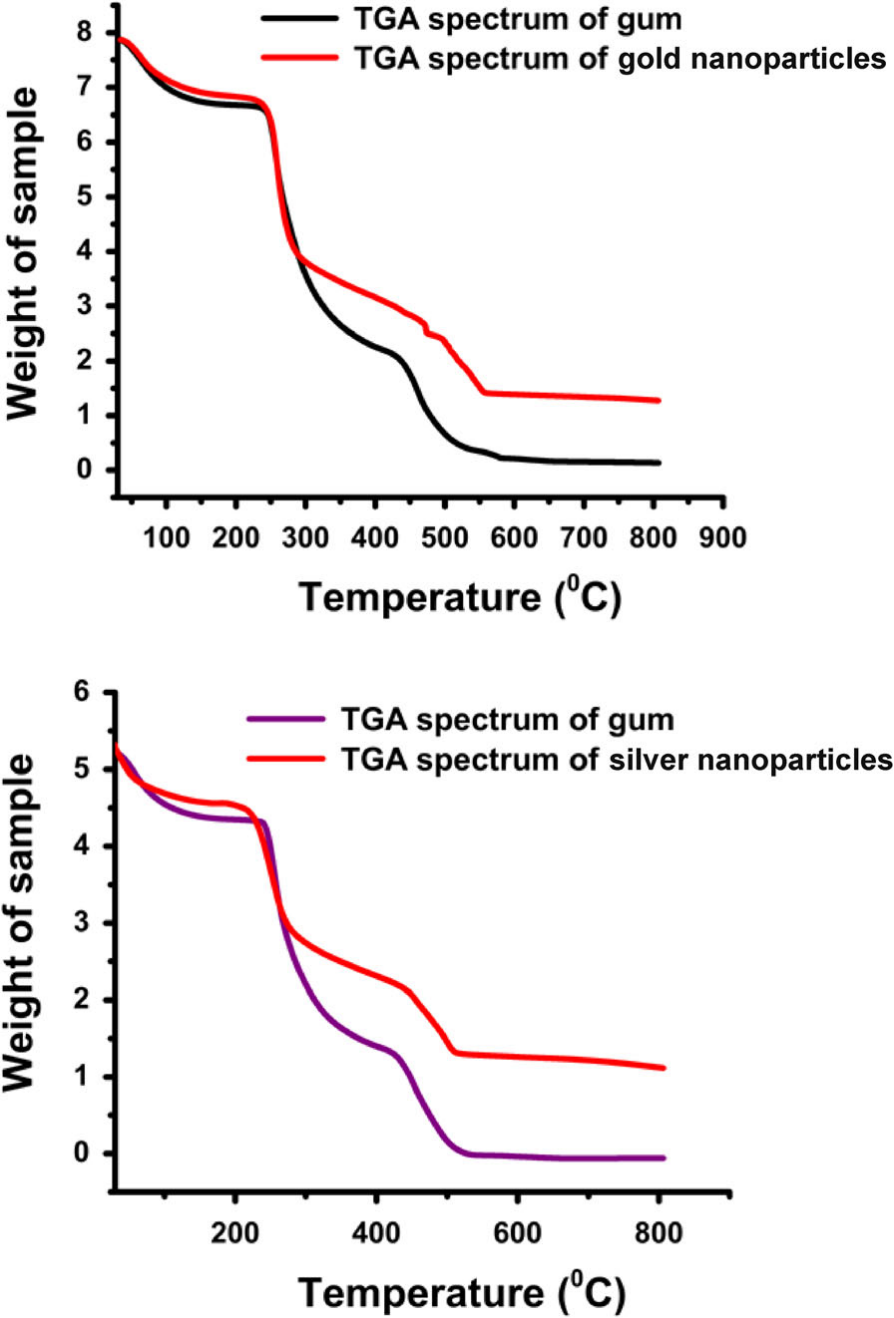
TGA spectra of *P. domestica* gum-loaded gold (**a**) and silver (**b**) nanoparticles

The thermogram obtained from the thermogravimetric analysis showed three successive weight losses for gum and nanoparticles within the temperature range of 50-800 °C (Fig. 6). The first observed weight loss was attributed to the loss of entrapped water molecules from the polymer matrix. The second weight loss might be due to the thermal decomposition of the polymer as well as the polymer capping around the nanoparticles. The third weight loss could be attributed to the conversion of remaining polymer to carbon residue. A low percent weight loss was observed for the gum after nanoparticle synthesis.

### Stability assessment

#### Effect of gum concentration

**Fig. 7:**
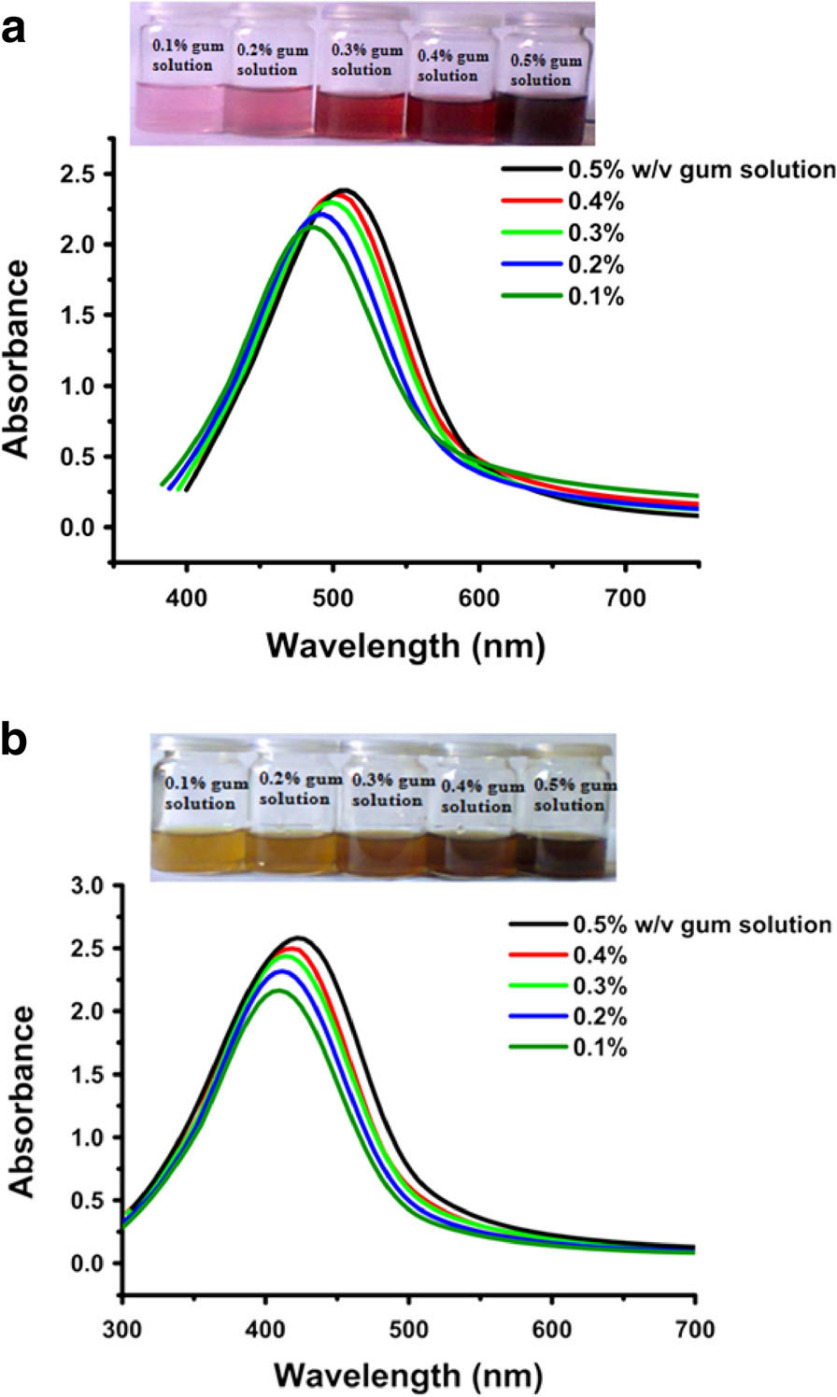
UV-Vis spectra showing the effect of gum concentration on the synthesis of *P. domestica* gum-loaded gold (**a**) and silver (**b**) nanoparticles. The inset photos show the corresponding color change of different solutions

An increase in the absorption intensity at 555 and 450 nm was observed, as the concentration of gum was increased. However, the characteristic SPR peaks for both the nanoparticles were slightly red shifted (Fig. 7). These changes indicate an enhancement in the nanoparticles concentration and an increase in the nanoparticle size. Both size and concentration of nanoparticles affect the absorbance. The concentration of gum affects the nanoparticle size distribution. The broad particle size distribution at high gum concentration, could be due to the increased intermolecular force of gum molecules, while, at lower gum concentration, it might be due to insufficient protection. In addition, a color change also indicates an increase in nanoparticle concentration due to increasing gum concentration. As concentration of gum was increased (0.1–0.5%), the colors of the obtained gold and silver nanoparticles were also intensified from light red to dark red and light yellow to dark yellow, respectively, indicating an increase in nanoparticle concentration.

#### Effect of Au (III) and Ag^+^ ions concentration

**Fig. 8:**
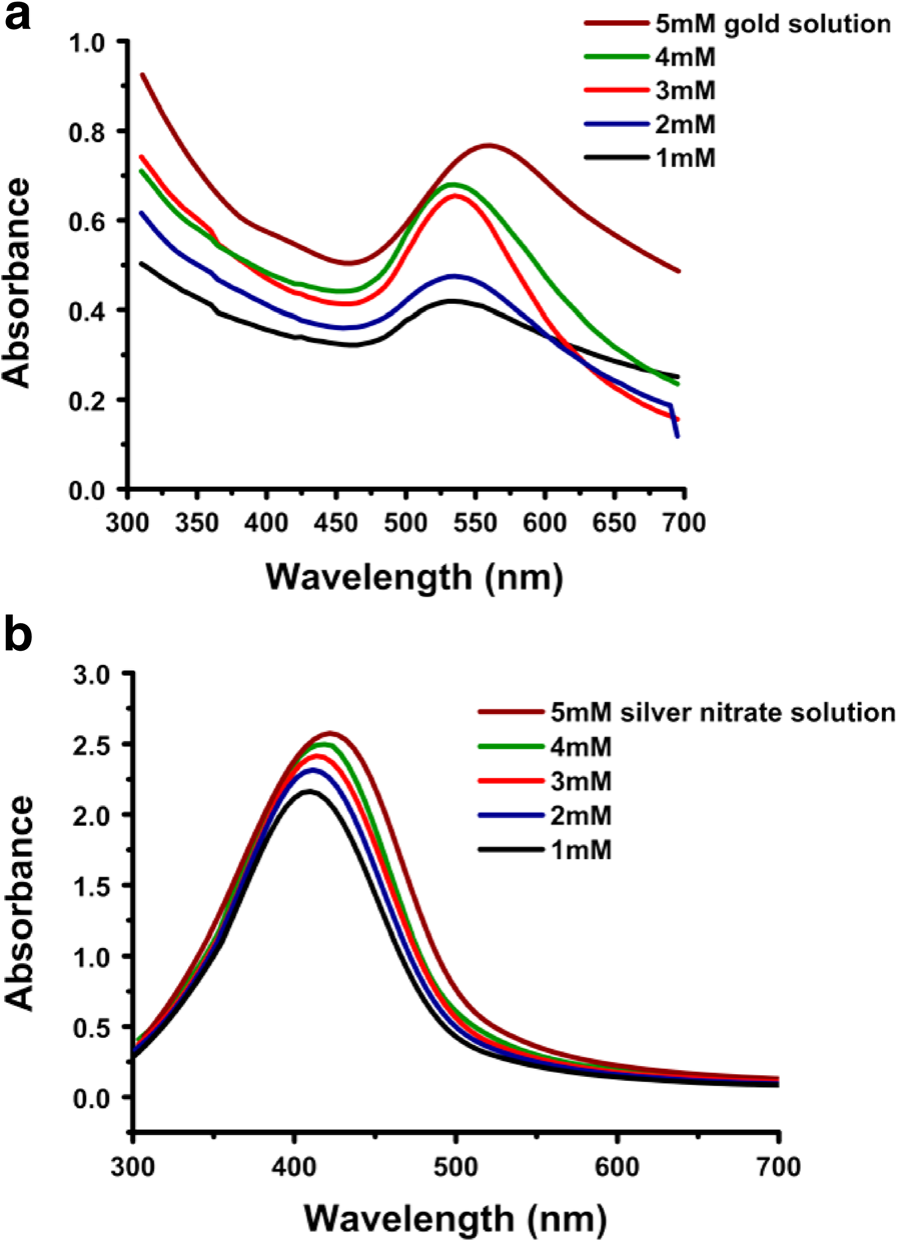
UV-Vis spectra showing the effect of metal ions concentration on the synthesis of *P. domestica* gum-loaded gold (**a**) and silver (**b**) nanoparticles

As indicated from the morphologies of the UV-Vis spectra (Fig. 8), an increase in the concentration of metal precursor, produced an increase in nanoparticles concentration, and a slight change in the particle shape and diameter. The increased number of colloidal particles in the gum network might be due to an increase in the number of metal nuclei with an increase in metal precursor concentration. The linear increase in the absorption intensity was due to an enhancement in the nuclei formation, which resulted in an increase in nanoparticle concentration, while the shift in absorption intensity can be attributed to an increase in the particle size. At high Au (III) and Ag^+^ ion concentrations (5 mM), quite larger particle size distributions were observed. This might be due to the less protective effects of the gum and account for the longitudinal plasmon resonance around 560 and 455 nm.

#### Effect of reaction temperature

**Fig. 9:**
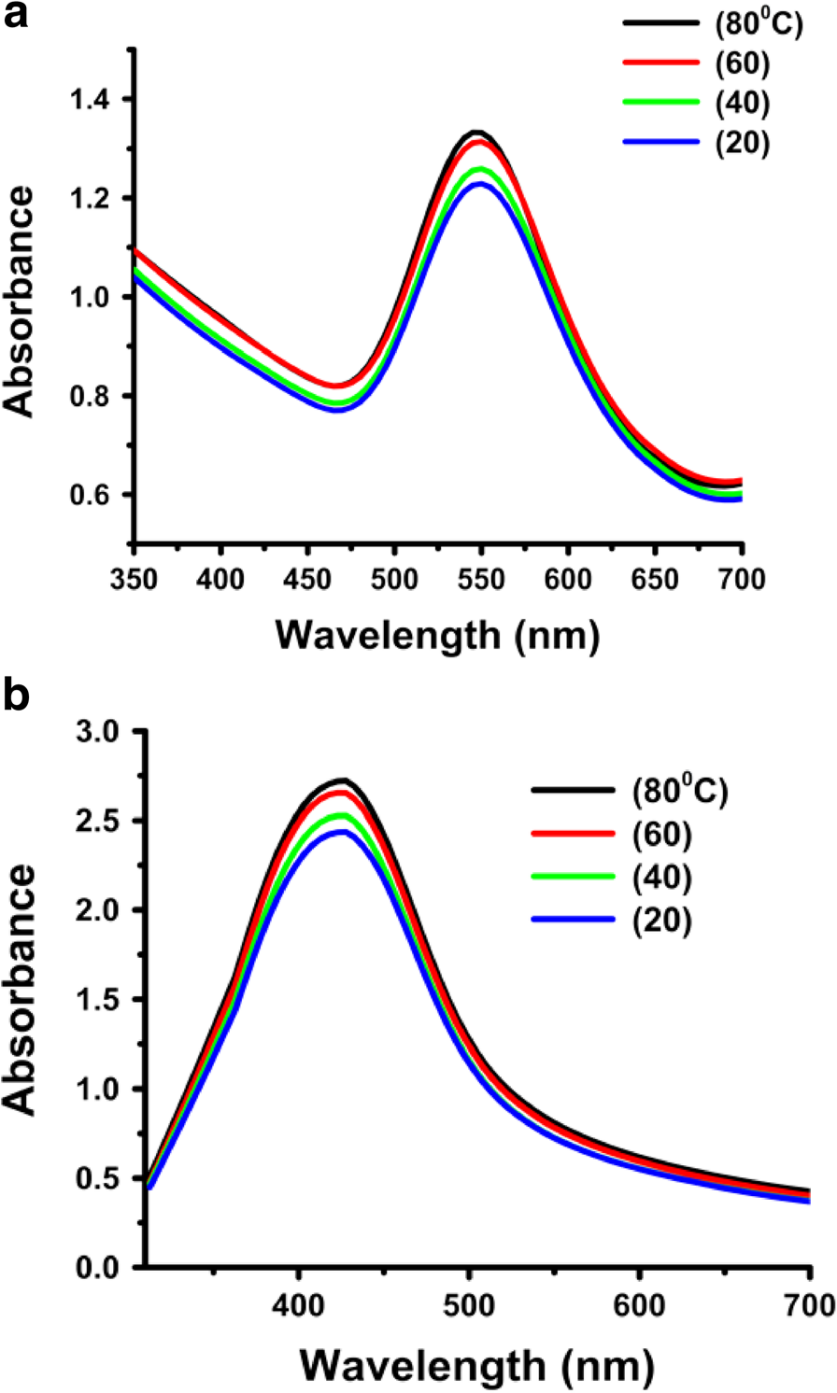
UV-Vis spectra showing the effect of reaction temperature on the synthesis of *P. domestica* gum-loaded gold (**a**) and silver (**b**) nanoparticles

With an increase in the reaction temperature, there was a linear increase in absorption intensity without any change in the position of the SPR peak (Fig. 9), indicating an increase in nanoparticle concentration of similar size. The maximum intensity of the characteristic absorption band was found at 80 °C, which is indicative of a maximum nanoparticle concentration and therefore the optimum temperature for nanoparticles synthesis.

#### Effect of reaction time

**Fig. 10:**
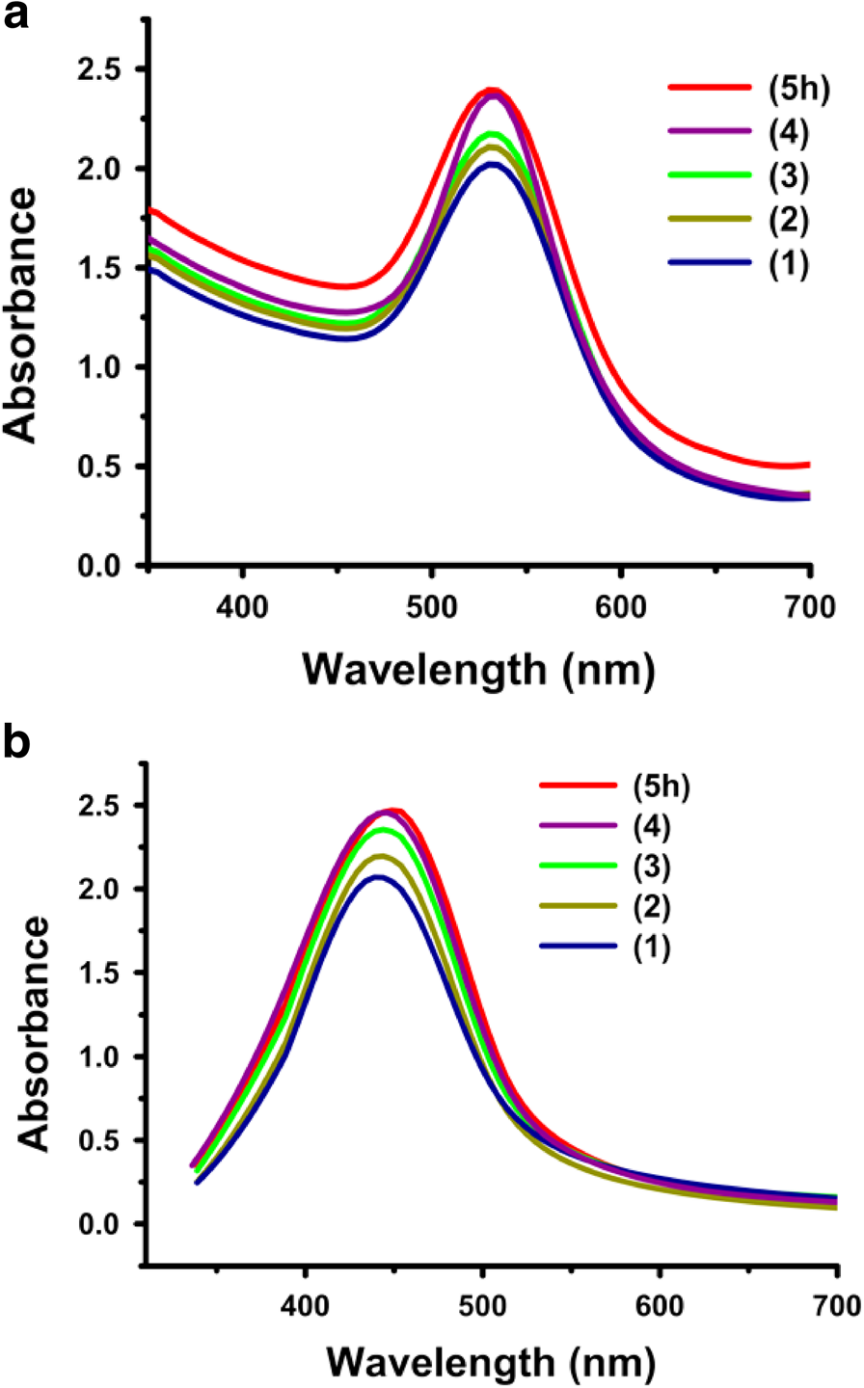
UV-Vis spectra showing the effect of reaction time on the synthesis of *P. domestica* gum-loaded gold (**a**) and silver (**b**) nanoparticles

An increase in the reduction capacity of the gum was observed with a change in the reaction time (1–5 h). The SPR peak occurs at 555 and 450 nm for gold and silver nanoparticles, respectively, and the intensity of absorption increases linearly without any shift in wavelength with time, thus indicating a continuous reduction of Au (III) and Ag^+^ ions, which led to an increase in the number of colloidal particles. However, after 4 h, no significant change in the intensity of the SPR peak was observed and is an indication of completion of the synthesis reaction (Fig. 10).

#### Effect of salt

**Fig. 11:**
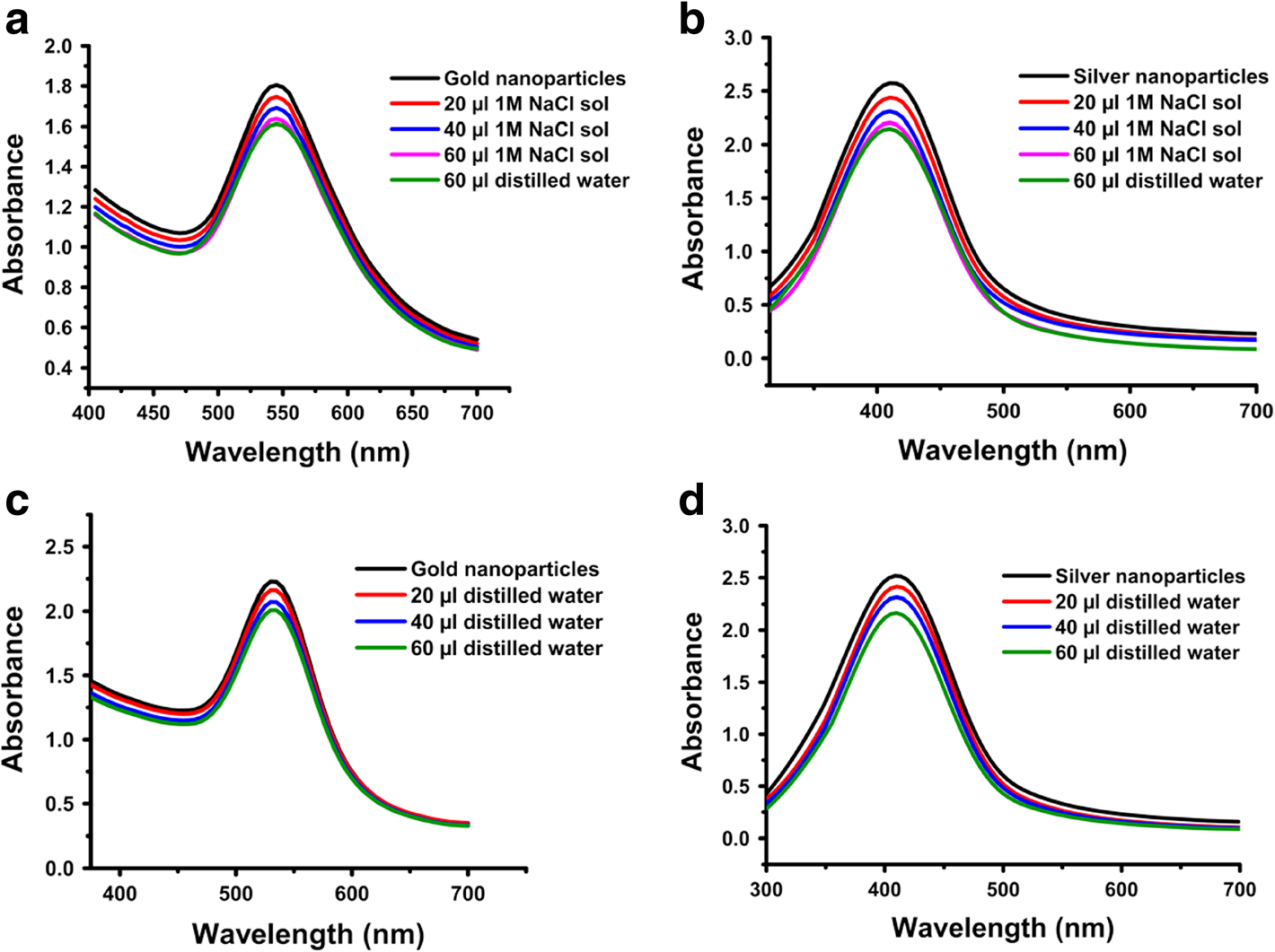
UV-Vis spectra showing the effect of different volumes of sodium chloride (NaCl) on *P. domestica* gum-loaded gold (**a**) and silver (**b**) nanoparticles, and the dilution effect of distilled water on *P. domestica* gum-loaded gold (**c**) and silver (**d**) nanoparticles

No obvious color change was observed, when different volumes of NaCl solutions (20, 40, and 60 μL) were added to the colloidal solutions of gold and silver nanoparticles. Moreover, no changes were observed in the position of SPR peaks with varying salt volumes. However, a decrease in their peak intensity was noted, which might be due to the dilution effect, as a similar decrease was also recorded for the salt-free nanoparticles solutions (Fig. 11).

#### Effect of pH

**Fig. 12:**
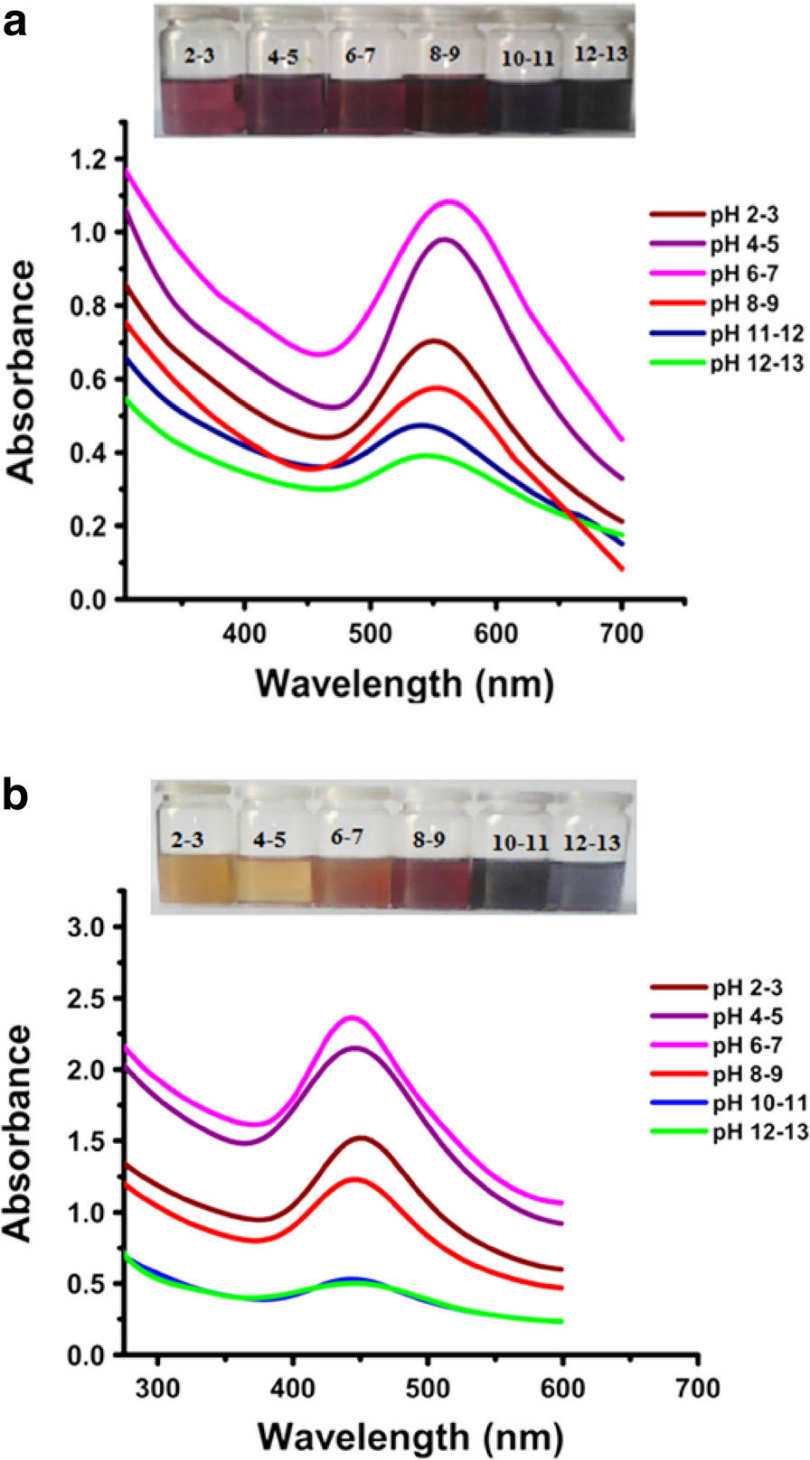
UV-Vis spectra showing the effect of different pH on *P. domestica* gum-loaded gold (**a**) and silver (**b**) nanoparticles

Optimized samples of both gold and silver nanoparticles are slightly acidic, having a pH value of 6–7, and showed maximum absorption in the UV-Vis spectra (Fig. 12). It was observed that the gold nanoparticles were stable in acidic media as there were less significant shifts in the UV-Vis absorption spectra except at pH 2–3, where the absorption intensity was lowered with a blue shift. The *P. domestica* gum-loaded gold nanoparticles were unstable in basic media, as significant peak lessening and blue shift were observed in their UV-Vis spectra. Similarly, the *P. domestica* gum-loaded silver nanoparticles also exhibited stability in neutral-to-acidic media; however, at pH 2–3, low absorption intensity was observed in the UV-Vis spectrum. In basic media, the silver nanoparticles were unstable except at pH 8–9, where the shift was similar to that of pH 2–3, but with low absorption intensity.

#### Storage stability

**Fig. 13:**
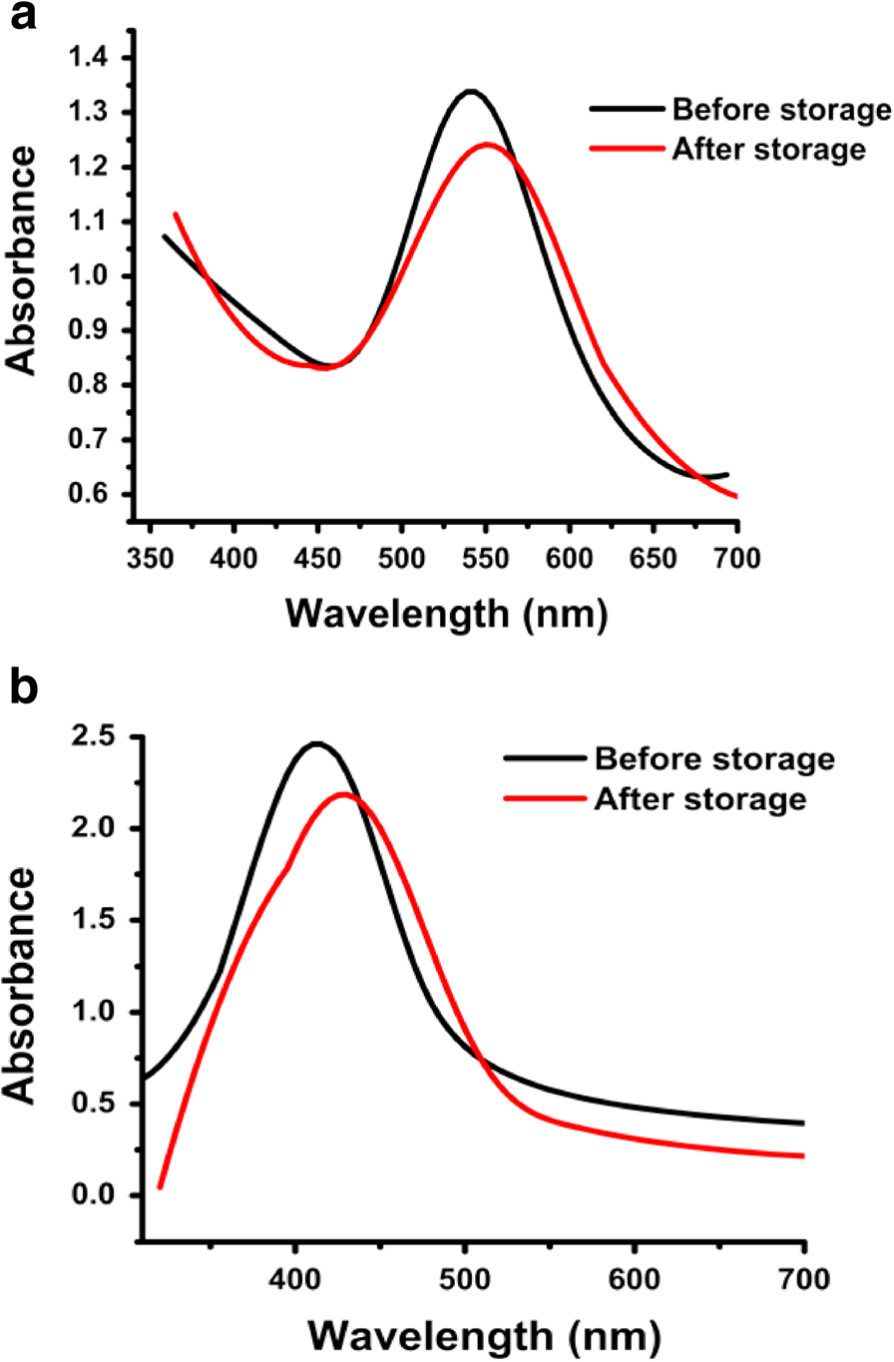
UV-Vis spectra showing the effect of long term storage on *P. domestica* gum-loaded gold (**a**) and silver (**b**) nanoparticles

*P. domestica* gum-loaded gold and silver nanoparticles solutions were stored at room temperature for six months. There was no change in color. Visual aggregation was observed during this storage period. From the UV-Vis spectra, a slight red shift was observed in the position of the SPR peak and a lessen absorption value, indicating an increase in the size of nanoparticles size due to aggregation (Fig. 13).

#### Thermal stability

**Fig. 14:**
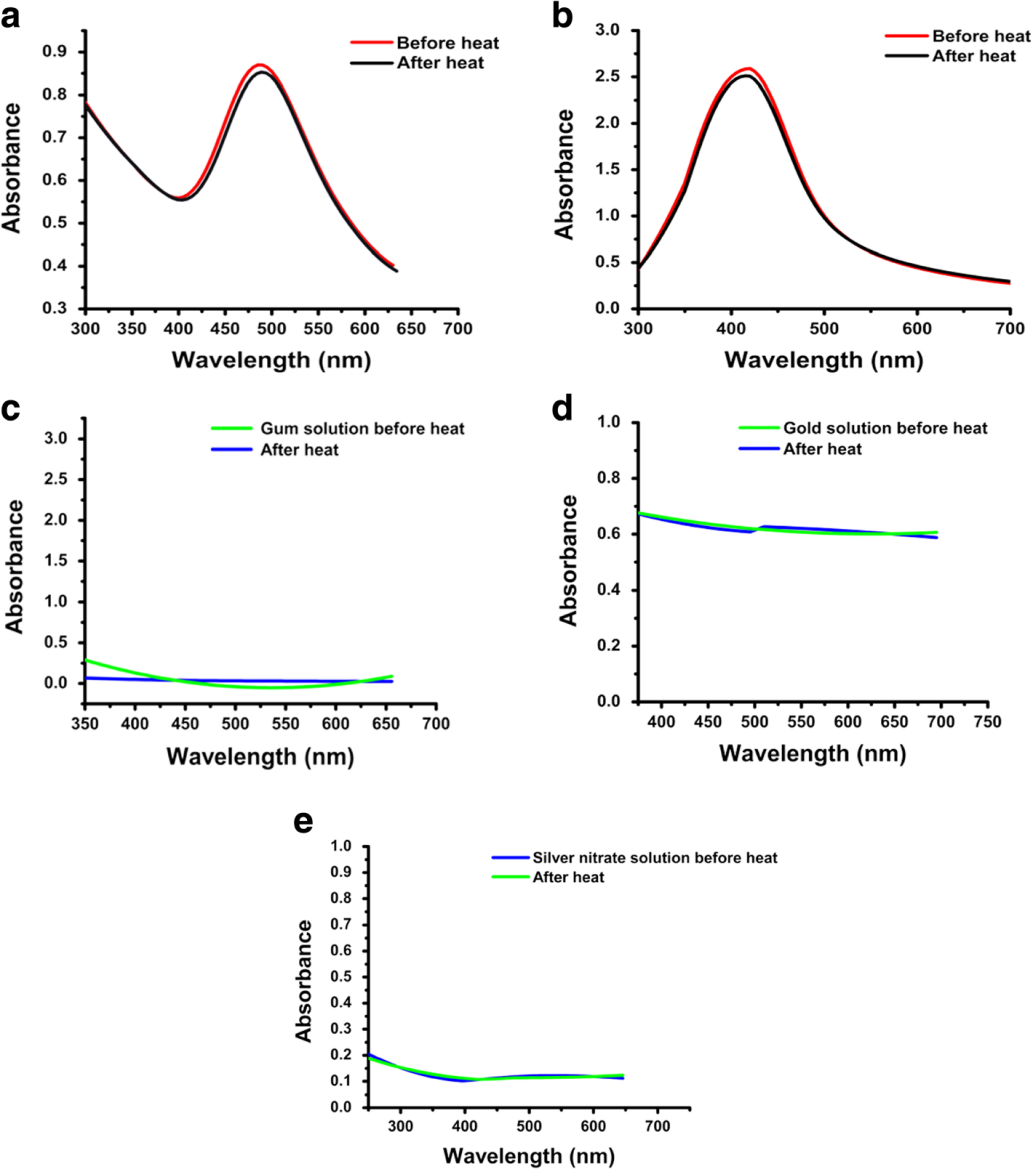
UV-Vis spectra showing the effect of extreme temperature (100 °C) on *P. domestica* gum-loaded gold (**a**) and silver (**b**) nanoparticles, *P. domestica* gum (**c**), gold solution (**d**), and silver nitrate solution (**e**)

Colloidal solutions of *P. domestica* gum-loaded gold and silver nanoparticles were heated at 100 °C for 30 min, and no significant changes were observed in the position of the SPR peaks (Fig. 14). This high stability of gold and silver nanoparticles can be reasonably attributed to the protective effect of biomolecules on the nanoparticle surface.

#### *Prunus domestica* gum-loaded nanoparticles inhibit cancer cells

*P. domestica* gum-loaded gold and silver nanoparticles were tested to evaluate their cytotoxic potential against human cervical cancer cells (HeLa) (Table 1). A potential anticancer effect was afforded by *P. domestica* gum-loaded gold nanoparticles, having an IC_50_ value of 2.14 ± 0.15 μg/mL, followed by *P. domestica* gum-loaded silver nanoparticles (3.45 ± 0.23 μg/mL) and *P. domestica* gum (4.92 ± 0.31 μg/mL). The standard anticancer drug cisplatin has the lowest IC_50_ value (1.89 ± 0.12), and therefore exhibited a robust inhibitory effect on the HeLa cancer cells.

**Table 1:** Anticancer activity of *Prunus domestica* gum-loaded gold and silver nanoparticles

Sample	IC_50_ (μg/mL)
*Prunus domestica* gum	4.92 ± 0.31
Gold nanoparticles	2.14 ± 0.15
Silver nanoparticles	3.45 ± 0.23
Cisplatin	1.89 ± 0.12

Values are expressed as mean ± SEM of three separate experiments

#### *Prunus domestica* gum-loaded nanoparticles suppressed pathogenic bacteria

The disk diffusion method was used to evaluate the potential antibacterial activity of *P. domestica* gum-loaded gold and silver nanoparticles against the pathological stains of Gram-positive and Gram-negative bacteria (Table 2). Prominent growth suppression was observed in plates loaded with nanoparticles, while the negative control plates and the *P. domestica* gum did not produce any visible zone of inhibition. The potential antibacterial effect was observed with *P. domestica* gum-loaded silver nanoparticles against Gram-positive strain of *S. aureus* (19.7 ± 0.4 mm) and Gram negative strains of *E. coli* (14.4 ± 0.7 mm), and *P. aeruginosa* (13.1 ± 0.2 mm). Similarly, these bacterial strains were also inhibited by *P. domestica* gum-loaded gold nanoparticles but with smaller zones of inhibition (10.5 ± 0.6 mm, 10 ± 0.4 mm and 8.2 ± 0.3 mm). The positive control, streptomycin also possessed an antibacterial effect of higher magnitude as compared to *P. domestica* gum-loaded gold and silver nanoparticles against the tested bacterial strains (23.6 ± 0.8 mm, 21.8 ± 0.2 mm and 18.6 ± 0.3 mm).

**Table 2:** Antibacterial activity of *Prunus domestica* gum-loaded gold and silver nanoparticles (zone of inhibition in millimeter)

Sample	*Staphylococcus aureus*	*Escherichia coli*	*Pseudomonas aeruginosa*
*Prunus domestica* gum	NA	NA	NA
Gold nanoparticles	10.5 ± 0.6	10 ± 0.4	8.2 ± 0.3
Silver nanoparticles	19.7 ± 0.4	14.4 ± 0.7	13.1 ± 0.2
Streptomycin	23.6 ± 0.8	21.8 ± 0.2	18.6 ± 0.3
DMSO negative control	NA	NA	NA

Values are expressed as mean ± SEM of three separate experiments. NA = Not active

#### *Prunus domestica* gum-loaded nanoparticles modify the enzymatic activity of urease

*P. domestica* gum-loaded gold and silver nanoparticles were tested in vitro for their potential effect as an inhibitor of urease. Both the gum and the loaded-nanoparticles were able to inhibit the jack-bean urease (Table 3). An increased inhibitory effect was demonstrated by the *P. domestica* gum (26.7 ± 0.49%), followed by *P. domestica* gum-loaded silver (21.5 ± 1.17%) and gold (19.2 ± 0.86%) nanoparticles. The positive control, thiourea showed a robust efficacy by producing a potent inhibition (78.3 ± 2.33%) of urease.

**Table 3:** Urease inhibition assay of *Prunus domestica* gum-loaded gold and silver nanoparticles

Sample	Percent inhibition (1 mg/mL)
*Prunus domestica* gum	26.7 ± 0.49
Gold nanoparticles	19.2 ± 0.86
Silver nanoparticles	21.5 ± 1.17
Thiourea (1 mM)	78.3 ± 2.33

Values are expressed as mean ± SEM of three separate experiments

#### *Prunus domestica* gum-loaded gold nanoparticles ameliorate inflammation

As shown in Table 4, the subplantar injection of carrageenan significantly elevated (*P* < 0.001) the normal paw volume. After 1 h, significant reduction in the carrageenan-induced paw edema was observed with *P. domestica* gum at doses of 200 mg/kg (*P* < 0.05) and 400 mg/kg (*P* < 0.001) as well as with the *P. domestica* gum-loaded gold nanoparticles at 40 mg/kg (*P* < 0.05) and 80 mg/kg (*P* < 0.01). Moreover, there was a significant antagonism (*P* < 0.001) of the phlogistic agent (carrageenan)-induced increase in paw volume by all the tested doses of *P. domestica* gum (200 and 400 mg/kg) and *P. domestica* gum-loaded gold nanoparticles (40 and 80 mg/kg) during the subsequent 2–5 h of experiment. A similar anti-inflammatory profile was shown by the positive control, diclofenac sodium, at a dose of 50 mg/kg during the 1st (*P* < 0.05) and 2–5 h (*P* < 0.001) of the study period.

**Table 4:** Anti-inflammatory activity of *Prunus domestica* gum-loaded gold nanoparticles

Treatment	1st h	2nd h	3rd h	4th h	5th h
Group 1 Saline	0.247 ± 0.026	0.250 ± 0.022	0.265 ± 0.013	0.250 ± 0.014	0.250 ± 0.016
Group 2 Carrageenan	0.355 ± 0.013^###^	0.370 ± 0.026^###^	0.385 ± 0.021^###^	0.397 ± 0.026^###^	0.405 ± 0.013^###^
Group 3 Diclofenac (50 mg/kg)	0.287 ± 0.029**	0.265 ± 0.019***	0.265 ± 0.031***	0.287 ± 0.025***	0.305 ± 0.012***
Group 4 Gum (200 mg/kg)	0.302 ± 0.009*	0.290 ± 0.008***	0.295 ± 0.013***	0.305 ± 0.013***	0.315 ± 0.019***
Group 5 Gum (400 mg/kg)	0.282 ± 0.017***	0.292 ± 0.012***	0.287 ± 0.012***	0.280 ± 0.022***	0.290 ± 0.018***
Group 6 Gold nanoparticles (40 mg/kg)	0.305 ± 0.021*	0.307 ± 0.012***	0.305 ± 0.012***	0.302 ± 0.015***	0.317 ± 0.026***
Group 7 Gold nanoparticles (80 mg/kg)	0.295 ± 0.013**	0.287 ± 0.017***	0.295 ± 0.013***	0.287 ± 0.022***	0.300 ± 0.018***

Values expressed as mean paw volume in mL **±** SD. ^**###**^ *P* <0.001 compared to group 1. * *P* <0.05, ** *P* <0.01, *** *P* <0.001 compared to group 2. One-way ANOVA followed by Tukey’s post hoc test. Results are the means of 6 animals per group

#### *Prunus domestica* gum-loaded gold nanoparticles de-escalate nociception

**Fig. 15:**
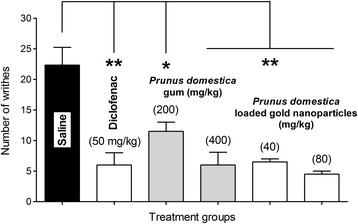
Antinociceptive effect of *P. domestica* gum-loaded gold nanoparticles. * *P* < 0.05, ** *P* < 0.01 compared to saline treated group. One-way ANOVA followed by Dunnett’s post hoc test. Results are the means of 6 animals per group

A potent antinocicpetive effect was demonstrated by the *P. domestica* gum and its gold nanoparticles (Fig. 15). Significant attenuation of chemically-induced nociception was observed with *P. domestica* gum at 200 (*P* < 0.05) and 400 mg/kg (*P* < 0.01). Similarly, the tested doses (40 and 80 mg/kg) of *P. domestica* gum-loaded gold nanoparticles significantly abolished (*P* < 0.01) the acetic acid-induced writhes. The single dose of the positive control, diclofenac sodium (50 mg/kg), also produced an efficient analgesic effect by significantly alleviating (*P* < 0.01) the chemically-induced nociceptive pain.

## Discussion

In this study, stabilized *Prunus domestica* gum-loaded gold and silver nanoparticles were evaluated for their multi-target therapeutic potential as an anticancer, antibacterial, urease inhibitor, anti-inflammatory, and antinociceptive agent. Nanoparticles can be loaded with a wide range of therapeutic agents, which together with a targeted delivery, increases the amount of drug accumulation at the pathological site, and decreases toxicity to normal tissues [30]. The use of natural gums for pharmaceutical applications is attractive as they are economical, readily available, nontoxic, capable of chemical modifications, potentially biodegradable, and also biocompatible. Natural gums have been widely employed as reducing, and stabilizing agents for the efficient synthesis of size-controlled nanoparticles that display greater stability, and better biological activities [31–33]. Phytotherapy offers new treatment opportunities as the therapeutic efficacy may based on the synergistic or antagonistic interaction of a mixture of phytochemicals [3]. Nano-based drug delivery systems can potentiate the action of plant extracts, promote sustained release of active constituents, reduce the required dose, decrease side effects, improve activity, and promote self-targeting at the infected pathological area [5].

*Prunus domestica* gum-loaded gold and silver nanoparticles were stable in different concentrations of NaCl (1–3 M), neutral to acidic pH (4–7) and did not show long-term storage (six months) or thermally (100 °C)-induced deterioration changes. Regarding the nanoparticles synthetic stability, 0.2 and 0.3% *w*/*v* gum, 3 and 4 mM Au/Ag ion solution, reaction temperature of 60-80 °C, and reaction time of 4 h were suitable for the efficient synthesis of *P. domestica* gum-loaded gold and silver nanoparticles. The biological utility of nanoparticles often stems from their unique physicochemical properties that are often a function of their surface interactions with other nanoparticles or biological entities. Therefore, apart from key attributes required for their successful use in biology, such as low toxicity, biocompatibility, and effective biological clearance, the colloidal or dispersion stability of these nanoparticles is also crucial to preserve their intended physicochemical behavior and hence their utility in the physiological environment [34].

Cancer remains a high unmet medical need. Conventional chemotherapy employs drugs that are known to kill cancer cells effectively; however, these cytotoxic drugs kill healthy cells in addition to tumor cells, leading to serious adverse side effects that limit their clinical effectiveness. Recently, single-target chemotherapy is fading in favor of a multi-target approach [35]. Coupling multiple targeted-agents or using an agent that hits an individual target in several independent locations in the disease-causing pathway(s) may be the best approach to treat different cancers [36]. In this study, the *P. domestica* gum-loaded gold and silver nanoparticles showed potential inhibitory propensity towards human cervical cancer cells (HeLa), and the anticancer effect was similar to that of the standard cisplatin. Nanotechnology can play an important role by allowing the appropriate combination of agents that act as “smart nanogrenades” by targeting cancer lesions and therefore eliminate them without collateral effects on healthy tissue [37]. Integration of nanocarriers as a novel drug delivery system in the traditional system of medicine can be proved beneficial to conflict chronic notorious diseases like cancer [38].

Infectious diseases remain among the leading cause of death and produce an extremely significant impact on global health and economies [39]. Infections caused by bacteria represent a major public health burden, not just in terms of morbidity and mortality, but also in terms of increased expenditure on patient management and implementation of infection control measures. Conventional drugs usually provide effective antibiotic therapy for bacterial infections, but there is an increasing problem of antibiotic resistance and a continuing need for new solutions. Immediate actions should be taken to counter the antibiotic resistance and reduce the development and spread of life-threatening infections [40]. Several herbal preparations with antibacterial activity have shown efficacy in different clinical trials against various pathogenic species of bacteria [41]. Our study shows that the *P. domestica* gum-loaded gold and silver nanoparticles possessed antibacterial properties, while the *P. domestica* gum itself was unable to produce any zone of inhibition. Preferential inhibition was demonstrated by the *P. domestica* gum-loaded silver nanoparticles against both the Gram-positive (*S. aureus*) and Gram-negative (*E. coi*, *P. aeruginosa*) pathogenic strains, and the effect was comparable to that of streptomycin, used as positive control. Nanotechnology can provide important tools for designing and fabricating a new generation of substrates with specific antimicrobial properties [42]. Herbal drugs incorporated into nano-based drug delivery systems have potential benefit for use in the treatment of different bacterial infections [43].

Ureases are metalloenzymes that hydrolyze urea into ammonia and carbon dioxide. Urease is a virulence factor found in various pathogenic bacteria and is essential for the host organism in the maintenance of bacterial cells in tissues [44]. The production of urease by *Helicobacter pylori*, a Gram-negative bacterium, plays a key role in protecting this bacterium from the fatal acidic environment of stomach. The colonization of *H. pylori* in the human stomach leads to gastric ulcer or even gastric carcinoma, if left untreated [45]. There is an urgent need of novel urease inhibitors for counteracting the catastrophe of *H. pylori* infection on the eve of rising antibiotic resistance. Our study showed that both the *P. domestica* gum and its gold nanoparticles possessed promising urease inhibitory potency. There is a great potential of plant secondary metabolites of different classes to negatively affect the activity of ureases, the knowledge of which can contribute to the design of novel, safe, and less-costly urease inhibitors with the aim to improve human life by fighting urease-related diseases [46]. Utilization of plant extract-fabricated nanoparticles to inhibit urease can be a boon for the development of new drugs to treat multidrug-resistant *H. pylori* by means of approaches such as their encapsulation with drugs or making synergistic combinations with standard drugs [47].

Inflammation is a complex set of interactions among soluble factors and cells that can arise in any tissue in response to traumatic, infectious, post-ischemic, toxic, or autoimmune injury. The process normally leads to recovery from infection and to healing; however, if targeted destruction and assisted repair are not properly phased, inflammation can lead to persistent tissue damage by leukocytes, lymphocytes, or collagen. Inflammation per se remains one of the main therapeutic targets in diverse disorders with a staggering collective impact [48]. Natural products play a significant role in human health in relation to the prevention and treatment of inflammatory conditions [49]. Inflammation induced by carrageenan is acute, nonimmune, and produces the cardinal signs of inflammation, i.e., edema, hyperalgesia, and erythema, which develop immediately following subcutaneous injection, resulting from the action of pro-inflammatory agents, including bradykinin, histamine, tachykinins, complement, and reactive oxygen, and nitrogen species. Such agents can be generated in situ at the site of insult or by infiltrating cells. Neutrophils readily migrate to sites of inflammation and can generate proinflammatory reactive oxygen and other species. The inflammatory response is usually quantified by an increase in paw size (edema), which is maximal around 5 h post-carrageenan injection and is modulated by inhibitors of specific molecules within the inflammatory cascade [29]. In this study, *P. domestica* gum (200 and 400 mg/kg) inhibited the carrageenan-induced biphasic paw edema response. Similarly, the *P. domestica* gum-loaded gold nanoparticles also exhibited a similar anti-inflammatory profile; however the beneficial effect was observed at much lower doses (40 and 80 mg/kg) compared to *P. domestica* gum, and the effect was comparable to that of the standard anti-inflammatory drug, diclofenac sodium. Current management of inflammatory processes can be improved by the use of biodegradable nanoplatforms that specifically deliver anti-inflammatory molecules to inflamed tissues [50]. Nanobiomaterials based on gold nanoparticles conjugated with biomolecules possessed unique anti-inflammatory properties by reducing the leukocyte-endothelium interaction and leukocyte influx to adjacent tissues after leukotriene B4 stimulation in vivo as well as producing a marked reduction of chemotaxis and oxidative burst activation in vitro [51].

Pain is an unpleasant sensory and emotional experience associated with actual or potential tissue damage or described in terms of such damage [52]. Pain is usually elicited by the activation of specific nociceptors (nociceptive pain). It may also result from injury to sensory fibers or from damage to the CNS itself (neuropathic pain) [53]. Writhing, which is an overt response to the intense pain induced by acetic acid through nociceptors, is characterized by episodes of retraction of the abdomen and stretching of the hind limbs. The signals transmitted to the central nervous system in response to pain due to irritation cause release of mediators, such as prostaglandins, which contribute to the increased sensitivity of nociceptors [54]. The acetic acid-induced nociceptive test is sensitive to analgesics [55], and sensory afferents in the peritoneum that carry various receptors on their terminals [56] are activated by appropriate agonists and therefore depress the generation of pain impulses [57, 58]. *P. domestica* gum has potential peripheral antinociceptive properties since a marked reduction in chemically-induced nociceptive response was noted at doses of 200 and 400 mg/kg. The extent to which *P. domestica* gum abolished the tonic visceral chemically-induced nociception was also exhibited by *P. domestica* gum-loaded gold nanoparticles. However, the effect was observed at much smaller doses (40 and 80 mg/kg) of gold nanoparticles and was similar to diclofenac sodium used as a standard analgesic. Chronic pain, resulting from disease or injury, constitutes an enormous burden for the individual and society. The effectiveness of current pain therapies is limited by the extent of pain relief provided and the occurrence of significant side effects. Nanotechnology has the potential to address multiple, major, unmet problems in the diagnosis, treatment, and symptom management of a large variety of diseases and conditions, such as cancer, which are accompanied by pain [59]. Gold nanoparticles have shown effectiveness to be useful for enhancing the analgesic effects of medicinal plant extracts with potential antinociceptive properties [60–62]. Nanomedicine offers unprecedented opportunities in the development of novel pain-relieving therapies that change the frowning face of pain to a smile of relief [63].

## Conclusions

*Prunus domestica* gum was effectively utilized to yield gold and silver nanoparticles, which maintained their nanoscale characteristics under a wide variety of simulated environmental and biological factors. The multi-target therapeutic potential of the *P. domestica* gum-loaded nanoparticles was explored in different in vitro and in vivo testing paradigms. Both the gold and silver nanoparticles possessed selective effects in inhibition of cancer cells, pathogenic bacteria, and urease. Additionally, the gum-loaded gold nanoparticles can limit medical conditions afflicted with inflammation and pain, which is due to their ability to ameliorate a phlogistic agent-induced paw edema and chemically-induced nociception, respectively. Nano-sized drug delivery systems of herbal drugs have the potential to impact multiple disease targets and thus present an advantage in control of complex disease systems compared to conventional therapeutics.

## Abbreviations

AgNO_3_: Silver nitrate
EDX: Energy dispersive X-ray spectroscopy
FTIR: Fourier transform infrared spectroscopy
HAuCl_4_.3H_2_O: Tetrachloroauric acid trihydrate
SEM: Scanning electron microscope
SPR: Surface plasmon resonance
TGA: Thermo gravimetric analysis
UV-Vis: Ultraviolet–visible
XRD: X-ray diffraction
